# Supplementary material for: ASSERT (Acute Sacral inSufficiEncy fractuRe augmenTation): randomised controlled, feasibility trial in older people
Source: BMJ Open. 2022 May 3;12(5):e050535. doi: 10.1136/bmjopen-2021-050535 (PMC9066477; doi:10.1136/bmjopen-2021-050535)
Supplement: Author's manuscript [file bmjopen-2021-050535.draft_revisions.pdf]

BMJ Open is committed to open peer review. As part of this commitment we make the peer review history of every article we publish publicly available.

When an article is published we post the peer reviewers' comments and the authors' responses online. We also post the versions of the paper that were used during peer review. These are the versions that the peer review comments apply to.

The versions of the paper that follow are the versions that were submitted during the peer review process. They are not the versions of record or the final published versions. They should not be cited or distributed as the published version of this manuscript.

BMJ Open is an open access journal and the full, final, typeset and author-corrected version of record of the manuscript is available on our site with no access controls, subscription charges or pay-per-view fees (http://bmjopen.bmj.com).

If you have any questions on BMJ Open's open peer review process please email info.bmjopen@bmj.com

BMJ Open

The ASSERT (Acute Sacral inSufficiEncy fractuRe augmenTation) Randomised Controlled, Feasibility Trial in Older People

Journal:	*BMJ Open*
Manuscript ID	bmjopen-2021-050535
Article Type:	Original research
Date Submitted by the Author:	23-Feb-2021
Complete List of Authors:	Ong, Terence; University of Malaya<br>Suazo Di Paola, Ana; University of Leicester, Leicester Clinical Trials Unit<br>Brookes, Cassandra; University of Leicester, Leicester Clinical Trials Unit<br>Drummond, Avril; University of Nottingham, Div of Rehabilitation and Ageing<br>Hendrick, Paul ; University of Nottingham, ; University of Nottingham,<br>Leighton, Paul; University of Nottingham, Division of Primary Care<br>Jones, Matthew; University of Nottingham, Division of Primay Care<br>Salem, Khalid; Nottingham University Hospitals NHS Trust, Centre for Spinal Studies and Surgery<br>Quraishi, Nasir; Nottingham University Hospitals NHS Trust, Centre for Spinal Studies and Surgery<br>Sahota, Opinder; Nottingham University Hospitals NHS Trust, HCOP
Keywords:	TRAUMA MANAGEMENT, PAIN MANAGEMENT, ORTHOPAEDIC & TRAUMA SURGERY, GERIATRIC MEDICINE

SCHOLARONE™
Manuscripts

I, the Submitting Author has the right to grant and does grant on behalf of all authors of the Work (as defined in the below author licence), an exclusive licence and/or a non-exclusive licence for contributions from authors who are: i) UK Crown employees; ii) where BMJ has agreed a CC-BY licence shall apply, and/or iii) in accordance with the terms applicable for US Federal Government officers or employees acting as part of their official duties; on a worldwide, perpetual, irrevocable, royalty-free basis to BMJ Publishing Group Ltd ("BMJ") its licensees and where the relevant Journal is co-owned by BMJ to the co-owners of the Journal, to publish the Work in this journal and any other BMJ products and to exploit all rights, as set out in our licence.

The Submitting Author accepts and understands that any supply made under these terms is made by BMJ to the Submitting Author unless you are acting as an employee on behalf of your employer or a postgraduate student of an affiliated institution which is paying any applicable article publishing charge ("APC") for Open Access articles. Where the Submitting Author wishes to make the Work available on an Open Access basis (and intends to pay the relevant APC), the terms of reuse of such Open Access shall be governed by a Creative Commons licence – details of these licences and which Creative Commons licence will apply to this Work are set out in our licence referred to above.

Other than as permitted in any relevant BMJ Author's Self Archiving Policies, I confirm this Work has not been accepted for publication elsewhere, is not being considered for publication elsewhere and does not duplicate material already published. I confirm all authors consent to publication of this Work and authorise the granting of this licence.

For peer review only

The ASSERT (Acute Sacral inSufficiEncy fractuRe augmenTation) Randomised Controlled, Feasibility Trial in Older People

Terence Ong[1], Ana Suazo Di Paola[2], Cassandra Brookes[2], Avril Drummond[3], Paul Hendrick[3], Paul Leighton[3], Matthew Jones[3], Khalid Salem[4], Nasir Quraishi[4], Opinder Sahota[3,4,5,6]

1. Faculty of Medicine, University of Malaya, Malaysia
2. Leicester Clinical Trials Unit. University of Leicester, UK
3. University of Nottingham, UK
4. The Centre for Spinal Studies and Surgery, Nottingham University Hospitals NHS Trust, UK
5. Depart HCOP, Nottingham University Hospitals NHS Trust, UK
6. National Institute for Health Research (NIHR) Nottingham Biomedical Research Centre (BRC), United Kingdom

Contact details:

Terence Ong
Department of Medicine,
Faculty of Medicine,
University of Malaya
50603 Kuala Lumpur
Malaysia

E-mail: terenceong@doctors.org.uk
Phone: +60128003827

For peer review only

The ASSERT (Acute Sacral inSufficiEncy fractuRe augmenTation) Randomised Controlled, Feasibility Trial in Older People

ABSTRACT

Objective: To determine the feasibility of designing and conducting a definitive trial to evaluate the effectiveness of sacral fracture fixation compared with non-surgical management among older people admitted with a lateral compression pelvic fragility fracture (PFF).

Design: Single site, parallel, two-arm randomised controlled feasibility trial.

Setting: A UK tertiary centre hospital

Participants: Patients aged ≥70 years who were ambulating pre-injury requiring hospital admission (within 28 days of injury) with a Type 1 lateral compression PFF.

Interventions: The intervention group received sacral fracture fixation (cement augmentation +/- screw fixation) within seven days of randomisation. Routine pre- and post-operative care followed each surgical intervention. The control group received usual care consisting of analgesia, and regular input from the medical and therapy team.

Primary and secondary outcome measures: The feasibility outcomes were the number of eligible patients, willingness to be randomised, adherence to allocated treatment, retention, data on the completeness and variability of the proposed definitive trial outcome measures, and reported adverse events.

Results: 241 patients were screened. 13 (5.4%) were deemed eligible to participate. Among the eligible participants, nine (69.2%) were willing to participate. Five participants were randomised to the intervention group and four to the control group. The clinicians involved were willing to allow their patients to be randomised and adhere to the allocated treatment. One participant in the intervention group and two participants in the control group received their allocated treatment. All participants were followed up until 12 weeks post-randomisation, and had an additional safety follow-up assessment at 12 months. Overall, the proportion of completeness of outcome measures was at least 75%. No adverse events were directly related to the trial.

Conclusions: There were significant challenges in recruiting sufficient participants which will need to be addressed prior to a definitive trial.

Trial registration: ISRCTN (reference number ISRCTN16719542).

Keywords: aged, sacral fracture, pelvic fracture, fragility fracture, hospital

For peer review only

The ASSERT (Acute Sacral inSufficiEncy fractuRe augmenTation) Randomised Controlled, Feasibility Trial in Older People

Strengths and limitations of this study

- This study highlighted the challenges in delivering a trial that would address the uncertainty of the role and timing of surgical intervention for acute sacral fractures.
- This feasibility study was designed to be pragmatic so that it could be delivered within current healthcare settings.
- This feasibility study was unable to report on the effectiveness of surgical fixation for sacral fractures.
- Only small number of participants fulfilled the eligibility criteria in this study and future trials need to address this.
- This study highlights the importance of conducting feasibility studies before undertaking large scale surgical studies in frail older people

For peer review only

The ASSERT (Acute Sacral inSufficiEncy fractuRe augmenTation) Randomised Controlled, Feasibility Trial in Older People

INTRODUCTION

Pelvic fragility fractures (PFF) are common and its incidence rises exponentially with age peaking in those aged 85 years and over [1-4]. Among older adults, it is mostly caused by falls and bone fragility due to osteoporosis [1,2]. Recent years have also seen the annual incidence of PFF rising and the absolute number of PFF hospitalised increased by 1.5 to 2 times [2-4]. The majority of these being older patients who require treatment in hospital to manage their pain and disability [1,3].

The most common PFF identified involves the pubic rami of the anterior pelvic ring [5,6]. However, 55-60% of these anterior pelvic ring fractures have concomitant involvement of the posterior ring, i.e., a sacral fracture [7,8]. The sacrum is the triangular base of the spine below the lumbar vertebras and forms the posterior part of the pelvic girdle [9]. Visualisation of sacral fractures on x-ray of the pelvis can be difficult [10,15]. Hence, many are diagnosed late when there is clinical suspicion of a more complex pelvic fracture [9,11]. Detection of posterior pelvic ring fractures is undertaken by either computerised tomography (CT) or magnetic resonance imaging (MRI) [12,13]. Such fractures that involve both the anterior and posterior part of the pelvic ring have worse outcomes. The average hospital length of stay for those with a combined anterior and posterior sacral fracture was on average two weeks longer than those with just an isolated anterior ring pubic rami fracture [8]; 30% more patients lose their previous independence permanently and the rate of institutionalisation is also higher [7].

The ultimate treatment goal for PFF is early restoration of mobility and function. This can only be achieved by effective and prompt pain relief. Fracture reduction and restoration of pelvic symmetry is less important. From a bio-mechanical point of view, an undisplaced anterior ring PFF is more stable than a posterior ring PFF. The pubic symphysis only contributes 15% towards pelvic stability compared to the posterior ring which provides the majority of the pelvis' structural support and stabilisation [14]. However, optimal pain control and early mobilisation remains challenging [15]. Around half of patients admitted with these fractures develop hospital and immobility complications [4,6,8,16]. One approach for treating such fractures is to stabilise the posterior ring fracture surgically and provide that potentially earlier pain relief, with a conservative, non-surgical approach for the more stable anterior pelvic ring fracture.

Surgical options for posterior ring fractures range from minimally invasive procedures, to open surgery with internal fixation [17-19]. Minimally invasive surgical techniques which involve percutaneous cement augmentation (injecting cement into the sacral ala at the side of the fracture) occasionally supplemented by a trans-sacral screw, also inserted using key-hole surgery, are increasingly being performed [20,21] and have been shown to reduce pain, reduce the amount of analgesia required post-operatively, increase patient mobility and are safe procedures in older people [12,22-24]. However, many of these studies were limited to observational and case-control studies which recruited a small number of participants and lacked a control arm.

A randomised controlled trial to evaluate the effectiveness of early surgical intervention for this type of pelvic fracture is required. Prior to conducting such a study, there remained uncertainty if such a trial could be delivered, the sample size required to determine its clinical effectiveness and the clinicians' adherence to allocated treatment groups. Hence, the aim of this present study was to determine the feasibility of a randomised controlled clinical trial of spinal sacral fixation (cement augmentation ± screw fixation) compared with current standard practice of non-surgical management among older people presenting to hospital with pubic rami and concomitant sacral fractures.

METHODS

A single-site, parallel, two-arm randomised controlled feasibility trial with participants allocated to either surgical or non-surgical intervention on a 1:1 ratio. Participants aged 70 years and over, ambulating with/without walking aids prior to their injury, admitted within 28 days of their injury and a Type 1 lateral compression (LC) pelvic fracture based on the Young-Burgess classification were invited to participate. The Young-Burgess classification is based on the predominant direction of the vector force at the time of injury. A Type 1 LC fracture involves an oblique or transverse pubic rami fracture and ipsilateral sacral compression fracture [25]. Fractures were confirmed either by CT or MRI imaging. Exclusion criteria were complex pelvic fractures (e.g., fractures involving / or close to the hip joint) requiring urgent surgery or progressive weight bearing exercises, pathological fracture in the context of known or suspected malignancy, previous surgery to the pelvis, any condition that precludes surgery or general/spinal anaesthesia, bedbound prior to the injury, receiving palliative care and clinically moribund on admission. During the start of the study, patients with a fracture that had occurred more than five days before hospital admission were also excluded. This was later amended to 28 days.

Participants had baseline data collected on recruitment and follow up assessments at weeks 2, 4, and 12 post-randomisation. All follow ups were done via a telephone interview except for week 2 where a face-to-face interview was conducted. Data was collected to assess the feasibility of this study and outcome measures for a future definitive trial. For the feasibility outcomes, information was gathered on the number of eligible patients, number of patients and doctors willing to be randomised, adherence to randomisation, rate of participant recruitment and retention, data on the completeness and variability of definitive trial outcome measures, failure of non-surgical care and adverse events in both arms. Outcome data collected for the definitive trial included: the timed up and go test (TUG) [26], Roland Morris Disability Questionnaire (RMDQ) [27], Montreal Cognitive Assessment (MoCA) [28], Functional Independence Measure (FIM) [29], Clinical Frailty Scale (CFS) [30], Charlson Comorbidity Index (CCI) [31], Barthel Activities of Daily Living (ADL) Index [32], Numeric Pain Rating Scale [33] and EuroQoL 5 Dimensions (EQ-5D-3L) score [34].

Participants were randomly allocated to either surgical intervention or non-surgical care (control group) via a secure web-based system (Sealed Envelope Ltd) by a member of the research team after completion of baseline data collection. The surgical team were informed of each participant's allocation. Those randomised to have surgery were assessed by a member of the surgical team for their suitability and choice of surgery based on the participant's general condition, fracture characteristics and surgeon's preference or experience. All surgery was planned to be carried out within 7 days post-randomisation. Pending surgery, participants received analgesia and had the required pre-operative tests. Participants randomised to the non-surgical arm would be started on appropriate analgesia and titrated accordingly. They also had input from the wider multidisciplinary team. If the participant's responsible medical team deemed there was a lack of response to non-surgical treatment, they could refer the participant to be considered for surgery. Participants who responded to analgesia while waiting for surgery would also have their indication for surgery re-assessed.

Sample size was calculated using data from another UK hospital of its pelvic fracture numbers [8]. A 10-month recruitment period was proposed, with the expectation to screen approximately 100 patients. Taking into account the assumption that 20% of patients screened would be ineligible, and that a 60% recruitment rate would be achieved during the recruitment period, it was then planned that a total of 48 participants would be recruited into the study. Furthermore, with an assumed 10% 3-month attrition rate, it was estimated that 43 participants would complete the study. If follow-up had been completed for these participants, it would have allowed the SD of the TUG to be estimated with an approximate SE of 1.2 assuming the SD is approximately 8 (95% CI: 6.6,10.2) and an SE of 0.9 for the RMDQ, assuming the SD is about 6 (95% CI: 4.9,7.6).

For peer review only

Participant characteristics and outcome data were reported using appropriate descriptive statistics by treatment arm and overall. The feasibility outcomes were also analysed descriptively. Outcomes were analysed on an intention-to-treat basis. This study received patient and public involvement (PPI) input through volunteer members of the Royal Osteoporosis Society's local support group. This study's PPI members had personal experience of PFFs and acknowledged the need to determine optimal hospital care and the potential role for surgery in these fractures. PPI members were part of the grant application. Focus groups with PPI members informed the design of the study and choice of study outcomes for the eventual definitive trial. All participant facing documents were reviewed by PPI members. They were present at each research meeting. Research ethics approval was granted by the North East; Newcastle and North Tyneside 2 research ethics committee (reference number 18/NE/0212). The study was registered on a clinical trials registry (https://www.isrctn.com, reference number ISRCTN16719542). The full protocol has been published [35]. Reporting of this study adhered to CONSORT reporting guidelines.

RESULTS

A total of 241 potential participants were screened over the recruitment period from 15.11.2018 to 31.07.2019. Among those screened, 13 (5.4%) were deemed eligible to take part in the study. The most frequent reasons for exclusion were because participants where either able to mobilise or had discharge plans made already (n=67), participants with complex fractures (n=35), participants with no sacral fracture (n=24), as well as participants whose injury occurred more than 5 days before their hospital admission (n=61, prior to amendment to eligibility criteria) (Figure 1).

Of the 13 eligible participants, nine (69.2%) consented to take part in the study (Figure 1). These participants sustained a combination of pelvic and sacral fractures after a fall from a standing height or less. A total of six participants randomised into the study had acute medical issues in addition to their PFF.

Five participants were randomised to the surgical treatment group and four to the non-surgical treatment group. One participant allocated to the surgical treatment group was subsequently withdrawn before receiving their allocated treatment as an exclusion criterion was identified post-randomisation. Four participants were allocated to each intervention group (Table 1). The clinical team and spinal surgical team were willing to randomise and adhere to the participant's treatment allocation. After subsequent assessments, only 1 participant (20%) in the surgical treatment group and 2 participants (50%) in the non-surgical treatment group received the allocated intervention.

Figure 1.

Demographic, baseline characteristics and procedural information of the participants recruited are detailed in table 1 (Table 1).

Table 1.

A total of three participants randomised into the study received surgical treatment regardless of their treatment allocation (one participant in the surgical treatment group and two participants in the non-surgical treatment group). The overall median time to operation was 6 days. All participants had cement augmentation. Data on any screws used were not available. Intra-operatively, one participant reported cement leakage and another one developed a respiratory problem.

The overall median (IQR) length of hospital stay corresponding to the eight participants taking part in the study was 10 (4.5, 19.5) days for those in the surgical treatment group and 7 (5.0, 23.0) days for

For peer review only

those in the non-surgical treatment group. Of the four participants in the surgical treatment group, two (50%) were discharged home without support and the remaining two (50%) to a rehabilitation facility. With regards to those in the non-surgical treatment group, one participant (25%) was discharged home with care assistance and the remaining three (75%) to a rehabilitation facility.

The overall proportion of completeness of outcome data collection at weeks 2, 4 and 12 was at least 75%. One participant was unable to take part in all the assessments corresponding to the Numeric Pain Rating Scale and the EQ-5D-3L Questionnaires due to cognitive impairment. Clinical outcomes are reported in Table 2.

Adverse events collected up to the 12 weeks follow-up time point were reported in 7 out of 8 participants (87.5%). None were related to the intervention provided for their fractures.

Table 2.

DISCUSSION

This feasibility study aimed to determine if a definitive clinical trial examining the role of spinal sacral fixation for sacral fractures and concomitant pubic rami was deliverable, as such a trial had never been conducted before. This study highlights the challenges of delivering such a trial on a larger scale. This study was unable to recruit adequate participants to meet the planned sample size. Despite active screening, the number of eligible participants that fulfilled the eligibility criteria was just over 5% (13 out of 241 screened). This study's eligibility criteria was as inclusive as possible, and through internal consensus, to reflect what would commonly be encountered in clinical practice.

Among those screened, almost 30% (67 out of 241 screened) were deemed clinically 'too well for surgery' by their medical team. They were able to mobilise with the analgesia prescribed and inpatient rehabilitation delivered by the multidisciplinary team. Of those eligible, approximately 31% (4 out of 13 eligible) declined to participate in the study, which was within what the study had anticipated. Although the treating clinicians and the surgical team were willing to randomise and adhere to the participants' allocated treatment, not all participants ultimately received the treatment they were allocated to. They were participants allocated to the surgical group where either their pain symptoms improved while waiting for surgery which negated the need for surgery or on further assessment the risk of surgery outweighed its potential benefit. The reverse was true for those allocated to the non-surgical group where despite optimal medical care, pain and disability persisted, and they were offered surgery.

Expanding the inclusion criteria to recruit those with only a sacral fracture could have potentially increased the number of participants recruited into the study. There may also have been patients with an acute pubic rami fracture but not had any further imaging done of the pelvis to detect further injuries. Only 46% of those admitted to hospital with a public rami fracture seen on plain radiograph underwent further imaging to visualise the entire pelvis [36]. At least half of pubic rami fractures have a concomitant posterior pelvic fracture but unless suspected by the clinician, this would either be missed or diagnosed late [7] thus, missing potential participants. Hence, an important requirement for such trials in the future is to embed detailed pelvic imaging in patients with a confirmed pubic rami fracture. Of the participants recruited, most were able to provide outcome measures for the required domains. Some assessment was limited by the presence of cognitive impairment. All participants were able to adhere to the follow up schedule.

This study was not designed to look at the effectiveness of surgical intervention compared to medical care. The data available was also unable to determine any trends or significant differences in outcomes between groups. However, this does not necessarily mean that there is no role for surgical intervention for older patients with these fractures. The non-randomised studies to date have

For peer review only

suggested a role for surgery in improving symptoms [12,22-24]. Additionally, the number of older people sustaining PFFs will increase and alongside it the healthcare utilisation to support them back to recovery. Surgical treatment may have a role in optimising recovery, similar to the role hip fracture fixation has in getting patients out of bed as early as the next day [36].

Hence, clinical trials are clearly required to understand the role, its effectiveness and timing of surgery in this group of patients. This was the first study that has looked at how best to design a trial to evaluate this. Issues around participant identification, eligibility, recruitment and understanding treatment decisions of hospital care still needs to be addressed before a definitive trial. Another feasibility study drawing on the challenges identified here is suggested. This would require an adequate sample size too. A single hospital site may not be able to achieve this and multi-site centres will be required. This study has emphasised the importance of feasibility studies as an important step prior to the delivery of complex clinical trials.

For peer review only

Funding

This study was funded by the National Institute for Health Research (NIHR) under its Research for Patient Benefit (RfPB) Programme (Grant Reference Number PB-PG-0816-20002).

Competing interests

The authors declare that they have no conflict of interest associated with this manuscript and its publication.

Author contribution

OS led the study design with contribution from all authors. Data collected were analysed by ASDP and CB. TO and OS drafted the manuscript. All authors read and approved the final manuscript.

Data availability statement

Data would be available from the authors on request.

Acknowledgement

The study team would like to acknowledge the input from the Healthcare of Older People research team who were involved in screening, recruitment and follow up of the study participants. The team would also want to thank the Patient and Public Involvement representatives, members of the Trial Steering Committee, Leicester Clinical Trials Unit, staff from the Department for Healthcare of Older People and Department for Spinal Surgery at Nottingham University Hospitals NHS Trust.

For peer review only

References

1. Prieto-Alhambra D, Aviles F.F, Judge A, Van Staa T, et al. Burden of pelvis fracture: a population-based study of incidence, hospitalisation and mortality. Osteoporos Int. 2012. 23:2797-2803.
2. Boufous S, Finch C, Lord S, Close J. The increasing burden of pelvic fractures in older people, New South Wales. Australia. Injury. 2005. 36:1323-1329.
3. Nanninga G, De Leur K, Panneman M.J.M, van der Elst M, Hartholt K.A. Increasing rate of pelvic fractures among older adults: The Netherlands, 1986-2011. Age Ageing. 2014. 43:648-653.
4. Buller L.T, Best M.J, Quinnan S.M. A nationwide analysis of pelvic ring fractures: Incidence and trends in treatment, length of stay and mortality. Geriatr Orthop Surg Rehabil. 2016. 7:9-17.
5. Morris R.O, Sonibare A, Green D.J, Masud T. Closed pelvic fractures: characteristics and outcomes in older patients admitted to medical and geriatric wards. Postgrad Med J. 2000. 76:646-650.
6. Taillandier J, Langue F, Alemanni M, Taillandier-Heriche E. Mortality and functional outcomes of pelvic insufficiency fractures in older patients. Joint Bone Spine. 2003. 70:287-289.
7. Studer P, Suhm N, Zappe B, Bless N, Jakob M. Pubic rami fractures in the elderly – a neglected injury. Swiss Med Wkly. 2013. 143:w13859.
8. Alnaib M, Waters S, Shanshal Y, Caplan N, et al. Combined pubic rami and sacral osteoporotic fractures: a prospective study. J Orthopaed Traumatol. 2012. 13:97-103.
9. Gupta A.C, Yoo A.J, Stone J, Barr J.C, et al. Percutaneous sacroplasty. J Neuro Intervent Surg. 2012. 4:385-389.
10. Gotis-Graham I, McGuigan L, Diamond T, Portek I, et al. Sacral insufficiency fractures in the elderly. J Bone Joint Surg (Br). 1994. 76-B:882-886.
11. Trouvin A.P, Alcaix D, Somon T, Zarnitsky C. Analgesic effect of sacroplasty in osteoporotic sacral fractures: A study of six cases. Joint Bone Spine. 2012. 79:500-503.
12. Onen M.R, Yuvruk E, Naderi S. Reliability and effectiveness of percutaneous sacroplasty in sacral insufficiency fractures. J Clin Neurosci. 2015. 22:1601-1608.
13. Kao F.C, Hsu Y.C, Liu P.H, Yeh L.R, Wang J.T, Tu Y.K. Osteoporotic sacral insufficiency fracture. An easily neglected disease in elderly patients. Medicine. 2017. 96:e9100.
14. Durkin A, Sagi C, Durham R, Flint L. Contemporary management of pelvic fractures. Am J Surg. 2006. 192:211-223.
15. Lyders E.M, Whitlow C.T, Baker M.D, Morris P.P. Imaging and treatment of sacral insufficiency fractures. Am J Neuroradiol. 2010. 31:201-210.
16. Lim P.N, Ooi L.J, Ong T, Neighbour C, Sahota O. Pelvic fragility fractures in older people admitted to hospital: the clinical burden. Eur Geriatr Med. 2019. 10:147-150.
17. Vanderschot P. Treatment options of pelvic and acetabular fractures in patients with osteoporotic bone. Injury. 2007. 38:497–508.
18. Grubor P, Milicevic S, Biscevic M, Tanjga R. Selection of treatment method for pelvic ring fractures. Med Arh. 2011. 65:278–282.
19. Rommens P.M, Wagner D, Hofmann A. Surgical management of osteoporotic pelvic fractures: a new challenge. Eur J Trauma Emerg Surg. 2012. 38:499–509.
20. Shuler T.E, Boone D.C, Gruen G.S, Peitzman A.B. Percutaneous iliosacral screw fixation: early treatment for unstable posterior pelvic ring disruptions. J Trauma. 1995. 38:453–8.
21. Schwabe P, Altintas B, Schaser K.D, Druschel C, et al. 3Dfluoroscopy navigated percutaneous screw fixation of acetabular fractures. J Orthop Trauma. 2014. 28:700–706.
22. Talmadge J, Smith K, Dykes T, Mittleider D. Clinical impact of sacroplasty on patient mobility. J Vasc Interv Radiol. 2014. 25:911–915.
23. Christof Hopf J, Christian F, Krieglstein C.F, Muller L.P, Koslowsky T.C. Percutaneous iliosacral screw fixation after osteoporotic posterior ring fractures of the pelvis reduces pain significantly in elderly patients. Injury. 2015. 46:1631–1636.
24. Dougherty R.W, McDonald J.S, Cho Y.W, Wald J.T, Thielen K.R, Kallmes D.F. Percutaneous sacroplasty using CT guidance for pain palliation in sacral insufficiency fractures. J Neuro Intervent Surg. 2014. 6:57-60.
25. Young J.W, Burgess A.R, Brumback R.J, et al. Pelvic fractures: value of plain radiography in early assessment and management. Radiology. 1986.160:445–51.

For peer review only

26. Shumway-Cook A, Brauer S, Woollacott M. Timed up and go test. Physical Therapy. 2000. 80. 896-903.
27. Roland M, Fairbank J. The Roland-Morris Disability Questionnaire and the Oswestry Disability Questionnaire. Spine. 2000. 25:3115-3124.
28. Nasreddine Z.S, Phillips N.A, Bedirian V, Charbonneau S, et al. The Montreal Cognitive Assessment, MoCA: A brief screening tool for mild cognitive impairment. J AM Geriatr Soc. 2005. 53:695-699.
29. Linacre J.M, Heinemann J.W, Wright B.D, Granger C.V, Hamilton B.B. The structure and stability of the functional independence measure. Arch Phys Med Rehabil. 1994. 75:127-132.
30. Rockwood K, Song X, MacKnight C, Bergman H, et al. A global clinical measure of fitness and frailty in elderly people. CMAJ. 2005. 173:489-495.
31. Charlson M.E, Pompei P, Ales K.L, MacKenzie C.R. A new method of classifying prognostic comorbidity in longitudinal studies: development and validation. J Chron Dis. 1987. 40:373-383.
32. Quinn T.J, Langhorne P, Stott D.J. Barthel Index for stroke trials. Stroke. 2011. 42:1146-1151.
33. Breivik H, Borchgrevink P.C, Allen S.M, Rosseland L.A, et al. Assessment of pain. Br J Anaesth. 2008. 101:17-24.
34. EuroQol Group. EuroQol--a new facility for the measurement of health-related quality of life. Health Policy. 1990. 16:199–208.
35. Van Berkel D, Ong T, Drummond A, Hendrick P, et al. ASSERT (Acute Sacral insufficiency fractuRe augmenTation) randomised controlled, feasibility in older people trial: a study protocol. BMJ Open. 2019. 9:e032111.
36. Sahota, O., van Berkel, D., Ong, T. et al. Pelvic fragility fractures—the forgotten osteoporotic fracture!. Osteoporos Int. 2021. https://doi.org/10.1007/s00198-021-05848-z.
37. Sheehan K.J, Goubar A, Almilaji O, Martin F.C, et al. Discharge after hip fracture surgery by mobilisation timing: secondary analysis of the UK National Hip Fracture Database. Age Ageing. 2020. doi.org/10.1093/ageing/afaa204.

For peer review only

Table 1. Demographics, baseline characteristics and procedural information of the participants recruited.

Characteristics	Surgical treatment group (n=5)	Non-surgical treatment group (n=4)
Age, median (IQR)[a] years	85 (83, 88)	85.5 (84, 89.5)
Female, n (%)	5 (100%)	4 (100%)
Charlson Co-morbidity Index (CCI), median (IQR)	0 (0, 1)	0.5 (0, 1)
Montreal Cognitive Assessment (MoCA), median (IQR)	23 (16, 23)	24 (22, 29)*
Clinical Frailty Scale (CFS), median (IQR)	6 (4, 6)^	3 (2.5, 5)
Prescribed strong opioids, n(%)	5 (100%)	4 (100%)
Concomitant acute medical issues, n (%)	4 (80%)	2 (50%)
Presence of delirium, n (%)	0 (0%)	1 (25%)

*Data from 3 participants
^Data from 4 participants
[a] Inter-quartile range

For peer review only

Table 2. Outcomes at follow up visits compared to baseline measurement.

Characteristics: Median (IQR)[a]	Surgical treatment group (n=4)				Non-surgical treatment group (n=4)			
	Baseline	Week 2	Week 4	Week 12	Baseline	Week 2	Week 4	Week 12
Time up and go (TUG), measured in seconds	-	47.2 (29.9, 88.6)	-	22.6 (16.7, 25.1)	-	53.7 (28.0, 210.0)*	-	19.9 (19.0, 47.8)*
Roland Morris Disability Questionnaire (RMDQ)	-	13 (11.0, 15.5)	14.5 (9.5, 15.5)	8.5 (4.5, 10.5)	-	17.0 (14.0, 22.0)*	12.0 (10.0, 20.0)*	10.5 (6.0, 14.0)
Functional Independence Measure (FIM)	77.5 (67.5, 88.0)	114 (91, 119)*	-	120.5 (114.5, 125.0)	77.0 (51.5, 92.5)	100 (57, 117)	-	115.0 (86.0, 120.0)
Barthel Activities of Daily Living	11 (9, 13)	15.0 (11.5, 18.0)	19 (16, 19)*	19.0 (18.5, 19.5)	9 (5.5, 14.0)	14.0 (7.0, 17.0)	18.0 (14.0, 19.0)*	18.5 (11.5, 20.0)
Numeric pain rating scale	10 (9, 10)	5 (4.0, 7.0)*	5.0 (3.5, 6.5)	4.5 (2.5, 6.0)	10 (8, 10)*	7 (6.0, 8.0)*	7.0 (3.0, 9.0)*	4.5 (4.0, 5.0)†

*Data from 3 participants
†Data from 2 participants
[a] Inter-quartile range

For peer review only

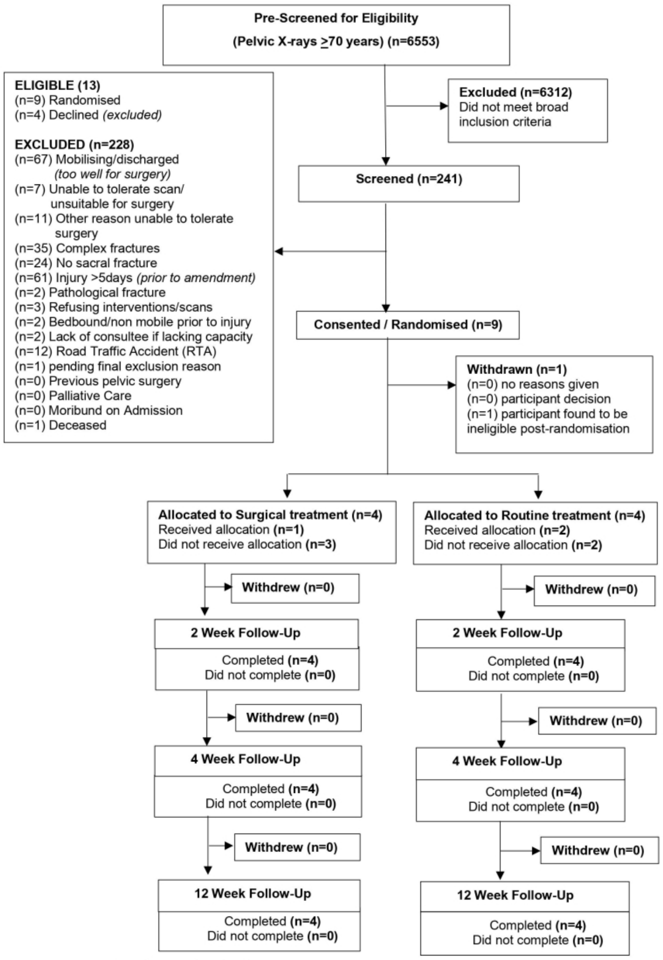

Figure 1. CONSORT diagram for the study.

CONSORT diagram for the study

209x297mm (300 x 300 DPI)

Reporting checklist for randomised trial.

Based on the CONSORT guidelines.

Instructions to authors

Complete this checklist by entering the page numbers from your manuscript where readers will find each of the items listed below.

Your article may not currently address all the items on the checklist. Please modify your text to include the missing information. If you are certain that an item does not apply, please write "n/a" and provide a short explanation.

Upload your completed checklist as an extra file when you submit to a journal.

In your methods section, say that you used the CONSORTreporting guidelines, and cite them as:

Schulz KF, Altman DG, Moher D, for the CONSORT Group. CONSORT 2010 Statement: updated guidelines for reporting parallel group randomised trials

		Reporting Item	Page Number
**Title and Abstract**			
Title	#1a	Identification as a randomized trial in the title.	1
Abstract	#1b	Structured summary of trial design, methods, results, and conclusions	1
**Introduction**			
Background and objectives	#2a	Scientific background and explanation of rationale	3
Background and objectives	#2b	Specific objectives or hypothesis	3
**Methods**			
Trial design	#3a	Description of trial design (such as parallel, factorial) including allocation ratio.	4
Trial design	#3b	Important changes to methods after trial commencement (such as eligibility criteria), with reasons	4

For peer review only

Participants	#4a	Eligibility criteria for participants	4
Participants	#4b	Settings and locations where the data were collected	4
Interventions	#5	The experimental and control interventions for each group with sufficient details to allow replication, including how and when they were actually administered	4
Outcomes	#6a	Completely defined prespecified primary and secondary outcome measures, including how and when they were assessed	4
Sample size	#7a	How sample size was determined.	4
Sample size	#7b	When applicable, explanation of any interim analyses and stopping guidelines	NA
Randomization - Sequence generation	#8a	Method used to generate the random allocation sequence.	
4			
Randomization - Sequence generation	#8b	Type of randomization; details of any restriction (such as blocking and block size)	
4			
Randomization - Allocation concealment mechanism	#9	Mechanism used to implement the random allocation sequence (such as sequentially numbered containers), describing any steps taken to conceal the sequence until interventions were assigned	NA
Randomization - Implementation	#10	Who generated the allocation sequence, who enrolled participants, and who assigned participants to interventions	4
Blinding	#11a	If done, who was blinded after assignment to interventions (for example, participants, care providers, those assessing outcomes) and how.	NA
Blinding	#11b	If relevant, description of the similarity of interventions	NA
Statistical methods	#12a	Statistical methods used to compare groups for primary and secondary outcomes	5
Statistical methods	#12b	Methods for additional analyses, such as subgroup analyses and adjusted analyses	NA

For peer review only

Outcomes	#6b	Any changes to trial outcomes after the trial commenced, with reasons	NA
**Results**			
Participant flow diagram (strongly recommended)	#13a	For each group, the numbers of participants who were randomly assigned, received intended treatment, and were analysed for the primary outcome	11
Participant flow	#13b	For each group, losses and exclusions after randomization, together with reason	11
Recruitment	#14a	Dates defining the periods of recruitment and follow-up	5
Recruitment	#14b	Why the trial ended or was stopped	NA
Baseline data	#15	A table showing baseline demographic and clinical characteristics for each group	12
Numbers analysed	#16	For each group, number of participants (denominator) included in each analysis and whether the analysis was by original assigned groups	12
Outcomes and estimation	#17a	For each primary and secondary outcome, results for each group, and the estimated effect size and its precision (such as 95% confidence interval)	5-6
Outcomes and estimation	#17b	For binary outcomes, presentation of both absolute and relative effect sizes is recommended	5-6
Ancillary analyses	#18	Results of any other analyses performed, including subgroup analyses and adjusted analyses, distinguishing pre-specified from exploratory	NA
Harms	#19	All important harms or unintended effects in each group (For specific guidance see CONSORT for harms)	5-6
**Discussion**			
Limitations	#20	Trial limitations, addressing sources of potential bias, imprecision, and, if relevant, multiplicity of analyses	6
Interpretation	#22	Interpretation consistent with results, balancing benefits and harms, and considering other relevant evidence	6
Registration	#23	Registration number and name of trial registry	5

For peer review only

Other Information

Protocol	#24	Where the full trial protocol can be accessed, if available	5
Funding	#25	Sources of funding and other support (such as supply of drugs), role of funders	8

The CONSORT checklist is distributed under the terms of the Creative Commons Attribution License CC-BY. This checklist was completed on 22. February 2021 using https://www.goodreports.org/, a tool made by the EQUATOR Network in collaboration with Penelope.ai

For peer review only

The ASSERT (Acute Sacral inSufficiEncy fractuRe augmenTation) Randomised Controlled, Feasibility Trial in Older People

Journal:	*BMJ Open*
Manuscript ID	bmjopen-2021-050535.R1
Article Type:	Original research
Date Submitted by the Author:	28-Feb-2022
Complete List of Authors:	Ong, Terence; Nottingham University Hospitals NHS Trust, Department of Healthcare for Older People; University of Malaya<br>Suazo Di Paola, Ana; University of Leicester, Leicester Clinical Trials Unit<br>Brookes, Cassandra; University of Leicester, Leicester Clinical Trials Unit<br>Drummond, Avril; University of Nottingham, Div of Rehabilitation and Ageing<br>Hendrick, Paul ; University of Nottingham, ; University of Nottingham,<br>Leighton, Paul; University of Nottingham, Division of Primary Care<br>Jones, Matthew; University of Nottingham, Division of Primay Care<br>Salem, Khalid; Nottingham University Hospitals NHS Trust, Centre for Spinal Studies and Surgery<br>Quraishi, Nasir; Nottingham University Hospitals NHS Trust, Centre for Spinal Studies and Surgery<br>Sahota, Opinder; Nottingham University Hospitals NHS Trust, HCOP
**Primary Subject Heading**:	Surgery
Secondary Subject Heading:	Surgery, Geriatric medicine
Keywords:	TRAUMA MANAGEMENT, PAIN MANAGEMENT, ORTHOPAEDIC & TRAUMA SURGERY, GERIATRIC MEDICINE

I, the Submitting Author has the right to grant and does grant on behalf of all authors of the Work (as defined in the below author licence), an exclusive licence and/or a non-exclusive licence for contributions from authors who are: i) UK Crown employees; ii) where BMJ has agreed a CC-BY licence shall apply, and/or iii) in accordance with the terms applicable for US Federal Government officers or employees acting as part of their official duties; on a worldwide, perpetual, irrevocable, royalty-free basis to BMJ Publishing Group Ltd ("BMJ") its licensees and where the relevant Journal is co-owned by BMJ to the co-owners of the Journal, to publish the Work in this journal and any other BMJ products and to exploit all rights, as set out in our licence.

The Submitting Author accepts and understands that any supply made under these terms is made by BMJ to the Submitting Author unless you are acting as an employee on behalf of your employer or a postgraduate student of an affiliated institution which is paying any applicable article publishing charge ("APC") for Open Access articles. Where the Submitting Author wishes to make the Work available on an Open Access basis (and intends to pay the relevant APC), the terms of reuse of such Open Access shall be governed by a Creative Commons licence – details of these licences and which Creative Commons licence will apply to this Work are set out in our licence referred to above.

Other than as permitted in any relevant BMJ Author's Self Archiving Policies, I confirm this Work has not been accepted for publication elsewhere, is not being considered for publication elsewhere and does not duplicate material already published. I confirm all authors consent to publication of this Work and authorise the granting of this licence.

For peer review only

The ASSERT (Acute Sacral inSufficiEncy fractuRe augmenTation) Randomised Controlled, Feasibility Trial in Older People

Terence Ong[1], Ana Suazo Di Paola[2], Cassandra Brookes[2], Avril Drummond[3], Paul Hendrick[3], Paul Leighton[3], Matthew Jones[3], Khalid Salem[4], Nasir Quraishi[4], Opinder Sahota[3,4,5,6]

1. Faculty of Medicine, University of Malaya, Malaysia
2. Leicester Clinical Trials Unit. University of Leicester, United Kingdom
3. University of Nottingham, United Kingdom
4. The Centre for Spinal Studies and Surgery, Nottingham University Hospitals NHS Trust, United Kingdom
5. Department of Healthcare for Older People, Nottingham University Hospitals NHS Trust, United Kingdom
6. National Institute for Health Research (NIHR) Nottingham Biomedical Research Centre (BRC), United Kingdom

Contact details:

Terence Ong
Department of Medicine,
Faculty of Medicine,
University of Malaya
50603 Kuala Lumpur
Malaysia

E-mail: terenceong@doctors.org.uk
Phone: +60128003827

For peer review only

The ASSERT (Acute Sacral inSufficiEncy fractuRe augmenTation) Randomised Controlled, Feasibility Trial in Older People

ABSTRACT

Objective: To determine the feasibility of designing and conducting a definitive trial to evaluate the effectiveness of sacral fracture fixation compared with non-surgical management among older people admitted with a lateral compression pelvic fragility fracture (PFF).

Design: Single site, parallel, two-arm randomised controlled feasibility trial.

Setting: A UK tertiary centre hospital

Participants: Patients aged ≥70 years who were ambulating pre-injury requiring hospital admission (within 28 days of injury) with a Type 1 lateral compression PFF.

Interventions: The intervention group received sacral fracture fixation (cement augmentation +/- screw fixation) within seven days of randomisation. Routine pre- and post-operative care followed each surgical intervention. The control group received usual care consisting of analgesia, and regular input from the medical and therapy team.

Primary and secondary outcome measures: The feasibility outcomes were the number of eligible patients, willingness to be randomised, adherence to allocated treatment, retention, data on the completeness and variability of the proposed definitive trial outcome measures, and reported adverse events.

Results: 241 patients were screened. 13 (5.4%) were deemed eligible to participate. Among the eligible participants, nine (69.2%) were willing to participate. Five participants were randomised to the intervention group and four to the control group. The clinicians involved were willing to allow their patients to be randomised and adhere to the allocated treatment. One participant in the intervention group and two participants in the control group received their allocated treatment. All participants were followed up until 12 weeks post-randomisation, and had an additional safety follow-up assessment at 12 months. Overall, the proportion of completeness of outcome measures was at least 75%. No adverse events were directly related to the trial.

Conclusions: There were significant challenges in recruiting sufficient participants which will need to be addressed prior to a definitive trial.

Trial registration: ISRCTN (reference number ISRCTN16719542).

Keywords: aged, sacral fracture, pelvic fracture, fragility fracture, hospital

For peer review only

The ASSERT (Acute Sacral inSufficiEncy fractuRe augmenTation) Randomised Controlled, Feasibility Trial in Older People

Strengths and limitations of this study

- This feasibility study was designed to be pragmatic so that it could be delivered within current healthcare setting.
- The inclusion criteria mirrored the group of patients where there is uncertainty of the role for surgical intervention.
- This feasibility study was unable to report on the effectiveness of surgical fixation for sacral fractures.

For peer review only

The ASSERT (Acute Sacral inSufficiEncy fractuRe augmenTation) Randomised Controlled, Feasibility Trial in Older People

INTRODUCTION

Pelvic fragility fractures (PFF) are common and its incidence rises exponentially with age peaking in those aged 85 years and over [1-4]. Among older adults, it is mostly caused by falls and bone fragility due to osteoporosis [1,2]. Recent years have also seen the annual incidence of PFF rising and the absolute number of PFF hospitalised increased by 1.5 to 2 times [2-4]. The majority of these being older patients who require treatment in hospital to manage their pain and disability [1,3].

The most common PFF identified involves the pubic rami of the anterior pelvic ring [5,6]. However, 55-60% of these anterior pelvic ring fractures have concomitant involvement of the posterior ring, i.e., a sacral fracture [7,8]. The sacrum is the triangular base of the spine below the lumbar vertebras and forms the posterior part of the pelvic girdle [9]. Visualisation of sacral fractures on x-ray of the pelvis can be difficult [10,11]. Hence, many are diagnosed late when there is clinical suspicion of a more complex pelvic fracture [9,11]. Detection of posterior pelvic ring fractures is undertaken by either computerised tomography (CT) or magnetic resonance imaging (MRI) [12,13]. Such fractures that involve both the anterior and posterior part of the pelvic ring have worse outcomes. The average hospital length of stay for those with a combined anterior and posterior sacral fracture was on average two weeks longer than those with just an isolated anterior ring pubic rami fracture [8]; 30% more patients lose their previous independence permanently and the rate of institutionalisation is also higher [7].

The ultimate treatment goal for PFF is early restoration of mobility and function. This can only be achieved by effective and prompt pain relief. Fracture reduction and restoration of pelvic symmetry is less important. From a bio-mechanical point of view, an undisplaced anterior ring PFF is more stable than a posterior ring PFF. The pubic symphysis only contributes 15% towards pelvic stability compared to the posterior ring which provides the majority of the pelvis' structural support and stabilisation [14]. However, optimal pain control and early mobilisation remains challenging [11,15]. Around half of patients admitted with these fractures develop hospital and immobility complications [4,6,8,16]. One approach for treating such fractures is to stabilise the posterior ring fracture surgically and provide that potentially earlier pain relief, with a conservative, non-surgical approach for the more stable anterior pelvic ring fracture.

Surgical options for posterior ring fractures range from minimally invasive procedures, to open surgery with internal fixation [17-19]. Minimally invasive surgical techniques which involve percutaneous cement augmentation (injecting cement into the sacral ala at the side of the fracture) occasionally supplemented by a trans-sacral screw, also inserted using key-hole surgery, are increasingly being performed [20,21] and have been shown to reduce pain, reduce the amount of analgesia required post-operatively, increase patient mobility and are safe procedures in older people [12,22-24]. However, many of these studies were limited to observational and case-control studies which recruited a small number of participants and lacked a control arm.

A randomised controlled trial to evaluate the effectiveness of early surgical intervention for this type of pelvic fracture is required. Prior to conducting such a study, there remained uncertainty if such a trial could be delivered, the sample size required to determine its clinical effectiveness and the clinicians' adherence to allocated treatment groups. Hence, the aim of this present study was to determine the feasibility of a randomised controlled clinical trial of spinal sacral fixation (cement augmentation ± screw fixation) compared with current standard practice of non-surgical management among older people presenting to hospital with pubic rami and concomitant sacral fractures.

For peer review only

METHODS

A single-site, parallel, two-arm randomised controlled feasibility trial with participants allocated to either surgical or non-surgical intervention on a 1:1 ratio. Participants aged 70 years and over, ambulating with/without walking aids prior to their injury, admitted within 28 days of their injury and a Type 1 lateral compression (LC) pelvic fracture based on the Young-Burgess classification were invited to participate. The Young-Burgess classification is based on the predominant direction of the vector force at the time of injury. A Type 1 LC fracture involves an oblique or transverse pubic rami fracture and ipsilateral sacral compression fracture [25]. Fractures were confirmed either by CT or MRI imaging. In the event of bilateral fractures, participants fulfilling the rest of the eligibility criteria would still be eligible for recruitment. Exclusion criteria were complex pelvic fractures (e.g., fractures involving / or close to the hip joint) requiring urgent surgery or progressive weight bearing exercises, pathological fracture in the context of known or suspected malignancy, previous surgery to the pelvis, any condition that precludes surgery or general/spinal anaesthesia, bedbound prior to the injury, receiving palliative care and clinically moribund on admission. During the start of the study, patients with a fracture that had occurred more than five days before hospital admission were also excluded. This was later amended to 28 days.

Participants had baseline data collected on recruitment and follow up assessments at weeks 2, 4, and 12 post-randomisation. All follow ups were done via a telephone interview except for week 2 where a face-to-face interview was conducted. Data was collected to assess the feasibility of this study and outcome measures for a future definitive trial. For the feasibility outcomes, information was gathered on the number of eligible patients, number of patients and doctors willing to be randomised, adherence to randomisation, rate of participant recruitment and retention, data on the completeness and variability of definitive trial outcome measures, failure of non-surgical care and adverse events in both arms. Outcome data collected for the definitive trial included: the timed up and go test (TUG) [26], Roland Morris Disability Questionnaire (RMDQ) [27], Montreal Cognitive Assessment (MoCA) [28], Functional Independence Measure (FIM) [29], Clinical Frailty Scale (CFS) [30], Charlson Comorbidity Index (CCI) [31], Barthel Activities of Daily Living (ADL) Index [32], Numeric Pain Rating Scale [33] and EuroQoL 5 Dimensions (EQ-5D-3L) score [34].

Participants were randomly allocated to either surgical intervention or non-surgical care (control group) via a secure web-based system (Sealed Envelope Ltd) by a member of the research team after completion of baseline data collection. The surgical team were informed of each participant's allocation. Those randomised to have surgery were assessed by a member of the surgical team for their suitability and choice of surgery based on the participant's general condition, fracture characteristics and surgeon's preference or experience. All surgery was planned to be carried out within 7 days post-randomisation. Pending surgery, participants received analgesia and had the required pre-operative tests. Participants randomised to the non-surgical arm would be started on appropriate analgesia and titrated accordingly. They also had input from the wider multidisciplinary team. If the participant's responsible medical team deemed there was a lack of response to non-surgical treatment, they could refer the participant to be considered for surgery. Participants who responded to analgesia while waiting for surgery would also have their indication for surgery re-assessed.

Sample size was calculated using data from another UK hospital of its pelvic fracture numbers [8]. A 10-month recruitment period was proposed, with the expectation to screen approximately 100 patients. Taking into account the assumption that 20% of patients screened would be ineligible, and that a 60% recruitment rate would be achieved during the recruitment period, it was then planned that a total of 48 participants would be recruited into the study. Furthermore, with an assumed 10% 3-month attrition rate, it was estimated that 43 participants would complete the study. If follow-up had been completed for these participants, it would have allowed the SD of the TUG to be estimated with an approximate SE of 1.2 assuming the SD is approximately 8 (95% CI: 6.6,10.2) and an SE of 0.9 for the RMDQ, assuming the SD is about 6 (95% CI: 4.9,7.6).

Participant characteristics and outcome data were reported using appropriate descriptive statistics by treatment arm and overall. The feasibility outcomes were also analysed descriptively. Outcomes were analysed on an intention-to-treat basis. Research ethics approval was granted by the North East;

For peer review only

Newcastle and North Tyneside 2 research ethics committee (reference number 18/NE/0212). The study was registered on a clinical trials registry (https://www.isrctn.com, reference number ISRCTN16719542). The full protocol has been published [35]. Reporting of this study adhered to CONSORT reporting guidelines.

Patient and public involvement
This study received patient and public involvement (PPI) input through volunteer members of the Royal Osteoporosis Society's local support group. This study's PPI members had personal experience of PFFs and were included in the grant application. Focus groups with members of the local support group were also conducted which informed the design of the study and choice of study outcomes for the trial. All participant facing documents were reviewed by PPI members. The PPI members were members of the Trial Management Group.

RESULTS

A total of 241 potential participants were screened over the recruitment period from 15.11.2018 to 31.07.2019. Among those screened, 13 (5.4%) were deemed eligible to take part in the study. The most frequent reasons for exclusion were because participants where either able to mobilise or had discharge plans made already (n=67), participants with complex fractures (n=35), participants with no sacral fracture (n=24), as well as participants whose injury occurred more than 5 days before their hospital admission (n=61, prior to amendment to eligibility criteria) (Figure 1).

Of the 13 eligible participants, nine (69.2%) consented to take part in the study (Figure 1). These participants sustained a combination of pelvic and sacral fractures after a fall from a standing height or less. A total of six participants randomised into the study had acute medical issues in addition to their PFF.

Five participants were randomised to the surgical treatment group and four to the non-surgical treatment group. One participant allocated to the surgical treatment group was subsequently withdrawn before receiving their allocated treatment as an exclusion criterion was identified post-randomisation. Four participants were allocated to each intervention group (Table 1). The clinical team and spinal surgical team were willing to randomise and adhere to the participant's treatment allocation. After subsequent assessments, only 1 participant (20%) in the surgical treatment group and 2 participants (50%) in the non-surgical treatment group received the allocated intervention.

Figure 1.

Demographic, baseline characteristics and procedural information of the participants recruited are detailed in table 1 (Table 1).

Table 1.

A total of three participants randomised into the study received surgical treatment regardless of their treatment allocation (one participant in the surgical treatment group and two participants in the non-surgical treatment group). The overall median time to operation was 6 days. All participants had cement augmentation. Data on any screws used were not available. Intra-operatively, one participant reported cement leakage and another one developed a respiratory problem.

The overall median (IQR) length of hospital stay corresponding to the eight participants taking part in the study was 10 (4.5, 19.5) days for those in the surgical treatment group and 7 (5.0, 23.0) days for

For peer review only

those in the non-surgical treatment group. Of the four participants in the surgical treatment group, two (50%) were discharged home without support and the remaining two (50%) to a rehabilitation facility. With regards to those in the non-surgical treatment group, one participant (25%) was discharged home with care assistance and the remaining three (75%) to a rehabilitation facility.

The overall proportion of completeness of outcome data collection at weeks 2, 4 and 12 was at least 75%. One participant was unable to take part in all the assessments corresponding to the Numeric Pain Rating Scale and the EQ-5D-3L Questionnaires due to cognitive impairment. Clinical outcomes are reported in Table 2.

Adverse events collected up to the 12 weeks follow-up time point were reported in 7 out of 8 participants (87.5%). None were related to the intervention provided for their fractures.

Table 2.

For peer review only

DISCUSSION

This feasibility study aimed to determine if a definitive clinical trial examining the role of spinal sacral fixation for sacral fractures and concomitant pubic rami was deliverable, as such a trial had never been conducted before. It was designed to be pragmatic in nature. Its eligibility criteria was inclusive to reflect what would commonly be encountered in clinical practice where the ideal management of these patients remains uncertain. However, the study highlighted the challenges of delivering such a trial on a larger scale. It was unable to recruit adequate participants to meet the planned sample size. Despite active screening, the number of eligible participants that fulfilled the eligibility criteria was just over 5% (13 out of 241 screened).

Among those screened, almost 30% (67 out of 241 screened) were deemed clinically ‘too well for surgery’ by their medical team. They were able to mobilise with the analgesia prescribed and inpatient rehabilitation delivered by the multidisciplinary team. This echoed what had been reported in existing literature where most patients admitted with such fractures would be non-operatively managed [19, 36]. Of those eligible in this study, approximately 31% (4 out of 13 eligible) declined to participate in the study, which was within what the study had anticipated. Although the treating clinicians and the surgical team were willing to randomise and adhere to the participants’ allocated treatment, not all participants ultimately received the treatment they were allocated to. They were participants allocated to the surgical group where either their pain symptoms improved while waiting for surgery which negated the need for surgery or on further assessment the risk of surgery outweighed its potential benefit. The reverse was true for those allocated to the non-surgical group where despite optimal medical care, pain and disability persisted, and they were offered surgery.

Expanding the inclusion criteria to recruit those with only a sacral fracture could have potentially increased the number of participants recruited into the study. There may also have been patients with an acute pubic rami fracture but not had any further imaging done of the pelvis to detect further injuries. Only 46% of those admitted to hospital with a public rami fracture seen on plain radiograph underwent further imaging to visualise the entire pelvis [37]. At least half of pubic rami fractures have a concomitant posterior pelvic fracture but unless suspected by the clinician, this would either be missed or diagnosed late [7] thus, missing potential participants. Hence, an important requirement for such trials in the future is to embed detailed pelvic imaging in patients with a confirmed pubic rami fracture. However, an argument could be made that if patients were already improving and becoming less symptomatic following their fracture, further imaging would be unlikely to alter the treatment plan in clinical practice. Of the participants recruited, most were able to provide outcome measures for the required domains. Some assessment was limited by the presence of cognitive impairment. All participants were able to adhere to the follow up schedule.

This study was not designed to look at the effectiveness of surgical intervention compared to medical care. The data available was also unable to determine any trends or significant differences in outcomes between groups. However, this does not necessarily mean that there is no role for surgical intervention for older patients with these fractures. The non-randomised studies to date have suggested a role for surgery in improving symptoms [12,22-24,36]. Additionally, the number of older people sustaining PFFs will increase and alongside it the healthcare utilisation to support them back to recovery. Surgical treatment may have a role in optimising recovery, similar to the role hip fracture fixation has in getting patients out of bed as early as the next day [37]. Hence, the need for randomised clinical trials to inform clinicians the likely role for surgery in PFFs. To date, it remains uncertain what patient, clinical or fracture characteristics that would benefit from surgery. A pelvic fracture specialist group have also put forward a different classification for pelvic fractures specifically for older people with low trauma pelvic fractures to support better stratification of patients for surgical or non-surgical management [38].

Hence, clinical trials are clearly required to understand the role, its effectiveness and timing of surgery in this group of patients. This was the first study that has looked at how best to design a trial to evaluate this. Issues around participant identification, eligibility, recruitment and understanding treatment decisions of hospital care still needs to be addressed before a definitive trial. Such an approach where a feasibility study is conducted before a definitive trial is becoming more common [39]. Feasibility studies with clear objectives of what aspect is being investigated, such as recruitment capability, data and outcome collection procedures, acceptability and suitability of the intervention or study procedure, evaluation of the resources to deliver the study, and participants response to the intervention, improves the design of a future trial [40]. This study was an important first study in defining the parameters for a definitive, complex trial. Moving forward, addressing the recruitment challenges identified here is needed. A single hospital site will not be able to achieve the required numbers. A multi-site centre study is needed. Creating a network of hospitals that provide pelvic fracture surgery in the UK may support delivering the numbers required for a definitive trial.

For peer review only

Funding
This study was funded by the National Institute for Health Research (NIHR) under its Research for Patient Benefit (RfPB) Programme (Grant Reference Number PB-PG-0816-20002).

Competing interests
The authors declare that they have no conflict of interest associated with this manuscript and its publication.

Author contribution
All authors contributed in accordance to ICMJE criteria for authorship. Conceptualization, T.O, A.S.D.P, C.B, A.D, P.H, P.L, M.J, K.S, N.Q, and O.S.; Methodology, T.O, A.S.D.P, C.B, A.D, P.H, P.L, M.J, K.S, N.Q, and O.S.; Formal Analysis, T.O, A.S.D.P, C.B, A.D, and O.S.; Investigation, T.O, K.S, N.Q, and O.S.; Data Curation, A.S.D.P, and C.B.; Writing – Original Draft Preparation, T.O, A.D, and O.S.; Writing – Review & Editing, T.O, A.S.D.P, C.B, A.D, P.H, P.L, M.J, K.S, N.Q, and O.S.; Project Administration, O.S.; Funding Acquisition, T.O, A.S.D.P, C.B, A.D, P.H, P.L, M.J, K.S, N.Q, and O.S.

Data availability statement
Data would be available from the authors on request.

Acknowledgement
The study team would like to acknowledge the input from the Healthcare of Older People research team who were involved in screening, recruitment and follow up of the study participants. The team would also want to thank the Patient and Public Involvement representatives, members of the Trial Steering Committee, Leicester Clinical Trials Unit, staff from the Department for Healthcare of Older People and Department for Spinal Surgery at Nottingham University Hospitals NHS Trust.

For peer review only

References

1. Prieto-Alhambra D, Aviles F.F, Judge A, Van Staa T, et al. Burden of pelvis fracture: a population-based study of incidence, hospitalisation and mortality. Osteoporos Int. 2012. 23:2797-2803.
2. Boufous S, Finch C, Lord S, Close J. The increasing burden of pelvic fractures in older people, New South Wales. Australia. Injury. 2005. 36:1323-1329.
3. Nanninga G, De Leur K, Panneman M.J.M, van der Elst M, Hartholt K.A. Increasing rate of pelvic fractures among older adults: The Netherlands, 1986-2011. Age Ageing. 2014. 43:648-653.
4. Buller L.T, Best M.J, Quinnan S.M. A nationwide analysis of pelvic ring fractures: Incidence and trends in treatment, length of stay and mortality. Geriatr Orthop Surg Rehabil. 2016. 7:9-17.
5. Morris R.O, Sonibare A, Green D.J, Masud T. Closed pelvic fractures: characteristics and outcomes in older patients admitted to medical and geriatric wards. Postgrad Med J. 2000. 76:646-650.
6. Taillandier J, Langue F, Alemanni M, Taillandier-Heriche E. Mortality and functional outcomes of pelvic insufficiency fractures in older patients. Joint Bone Spine. 2003. 70:287-289.
7. Studer P, Suhm N, Zappe B, Bless N, Jakob M. Pubic rami fractures in the elderly – a neglected injury. Swiss Med Wkly. 2013. 143:w13859.
8. Alnaib M, Waters S, Shanshal Y, Caplan N, et al. Combined pubic rami and sacral osteoporotic fractures: a prospective study. J Orthopaed Traumatol. 2012. 13:97-103.
9. Gupta A.C, Yoo A.J, Stone J, Barr J.C, et al. Percutaneous sacroplasty. J Neuro Intervent Surg. 2012. 4:385-389.
10. Gotis-Graham I, McGuigan L, Diamond T, Portek I, et al. Sacral insufficiency fractures in the elderly. J Bone Joint Surg (Br). 1994. 76-B:882-886.
11. Lyders E.M, Whitlow C.T, Baker M.D, Morris P.P. Imaging and treatment of sacral insufficiency fractures. Am J Neuroradiol. 2010. 31:201-210.
12. Onen M.R, Yuvruk E, Naderi S. Reliability and effectiveness of percutaneous sacroplasty in sacral insufficiency fractures. J Clin Neurosci. 2015. 22:1601-1608.
13. Kao F.C, Hsu Y.C, Liu P.H, Yeh L.R, Wang J.T, Tu Y.K. Osteoporotic sacral insufficiency fracture. An easily neglected disease in elderly patients. Medicine. 2017. 96:e9100.
14. Durkin A, Sagi C, Durham R, Flint L. Contemporary management of pelvic fractures. Am J Surg. 2006. 192:211-223.
15. Trouvin A.P, Alcaix D, Somon T, Zarnitsky C. Analgesic effect of sacroplasty in osteoporotic sacral fractures: A study of six cases. Joint Bone Spine. 2012. 79:500-503.
16. Lim P.N, Ooi L.J, Ong T, Neighbour C, Sahota O. Pelvic fragility fractures in older people admitted to hospital: the clinical burden. Eur Geriatr Med. 2019. 10:147-150.
17. Vanderschot P. Treatment options of pelvic and acetabular fractures in patients with osteoporotic bone. Injury. 2007. 38:497–508.
18. Grubor P, Milicevic S, Biscevic M, Tanjga R. Selection of treatment method for pelvic ring fractures. Med Arh. 2011. 65:278–282.
19. Rommens P.M, Wagner D, Hofmann A. Surgical management of osteoporotic pelvic fractures: a new challenge. Eur J Trauma Emerg Surg. 2012. 38:499–509.
20. Shuler T.E, Boone D.C, Gruen G.S, Peitzman A.B. Percutaneous iliosacral screw fixation: early treatment for unstable posterior pelvic ring disruptions. J Trauma. 1995. 38:453–8.
21. Schwabe P, Altintas B, Schaser K.D, Druschel C, et al. 3Dfluoroscopy navigated percutaneous screw fixation of acetabular fractures. J Orthop Trauma. 2014. 28:700–706.
22. Talmadge J, Smith K, Dykes T, Mittleider D. Clinical impact of sacroplasty on patient mobility. J Vasc Interv Radiol. 2014. 25:911–915.
23. Christof Hopf J, Christian F, Krieglstein C.F, Muller L.P, Koslowsky T.C. Percutaneous iliosacral screw fixation after osteoporotic posterior ring fractures of the pelvis reduces pain significantly in elderly patients. Injury. 2015. 46:1631–1636.
24. Dougherty R.W, McDonald J.S, Cho Y.W, Wald J.T, Thielen K.R, Kallmes D.F. Percutaneous sacroplasty using CT guidance for pain palliation in sacral insufficiency fractures. J Neuro Intervent Surg. 2014. 6:57-60.
25. Young J.W, Burgess A.R, Brumback R.J, et al. Pelvic fractures: value of plain radiography in early assessment and management. Radiology. 1986.160:445–51.

For peer review only

26. Shumway-Cook A, Brauer S, Woollacott M. Timed up and go test. Physical Therapy. 2000. 80. 896-903.
27. Roland M, Fairbank J. The Roland-Morris Disability Questionnaire and the Oswestry Disability Questionnaire. Spine. 2000. 25:3115-3124.
28. Nasreddine Z.S, Phillips N.A, Bedirian V, Charbonneau S, et al. The Montreal Cognitive Assessment, MoCA: A brief screening tool for mild cognitive impairment. J AM Geriatr Soc. 2005. 53:695-699.
29. Linacre J.M, Heinemann J.W, Wright B.D, Granger C.V, Hamilton B.B. The structure and stability of the functional independence measure. Arch Phys Med Rehabil. 1994. 75:127-132.
30. Rockwood K, Song X, MacKnight C, Bergman H, et al. A global clinical measure of fitness and frailty in elderly people. CMAJ. 2005. 173:489-495.
31. Charlson M.E, Pompei P, Ales K.L, MacKenzie C.R. A new method of classifying prognostic comorbidity in longitudinal studies: development and validation. J Chron Dis. 1987. 40:373-383.
32. Quinn T.J, Langhorne P, Stott D.J. Barthel Index for stroke trials. Stroke. 2011. 42:1146-1151.
33. Breivik H, Borchgrevink P.C, Allen S.M, Rosseland L.A, et al. Assessment of pain. Br J Anaesth. 2008. 101:17-24.
34. EuroQol Group. EuroQol--a new facility for the measurement of health-related quality of life. Health Policy. 1990. 16:199–208.
35. Van Berkel D, Ong T, Drummond A, Hendrick P, et al. ASSERT (Acute Sacral insufficiency fractuRe augmenTation) randomised controlled, feasibility in older people trial: a study protocol. BMJ Open. 2019. 9:e032111.
36. Rommens P.M, Hopf J.C, Herteleer M, Devlieger B, Hofman A, Wagner D. Isolated pubic ramus fractures are serious adverse events for elderly persons: An observational study on 138 patients with fragility fractures of the pelvic Type 1 (FFP Type 1). J Clin Med. 2020. 9:2498.
37. Sahota, O., van Berkel, D., Ong, T. et al. Pelvic fragility fractures—the forgotten osteoporotic fracture!. Osteoporos Int. 2021. https://doi.org/10.1007/s00198-021-05848-z.
38. Romens P.M, Wagner D, Hofman A. Do we need a separate classification for fragility fractures of the pelvic. J Orthop Trauma. 2019. 33:S55-S60.
39. Skivington K, Matthews L, Simpson S.A, Craig P, Baird J, Blazeby J.M, et al. A new framework for developing and evaluating complex interventions: update of Medical Research Council guidance. BMJ. 2021. 374:n2061.
40. Orsmond G.I, Cohn E.S. The distinctive features of a feasibility study: objectives and guiding questions. OTJR (Thorofare N J). 2015. 35:169-177.

For peer review only

Table 1. Demographics, baseline characteristics and procedural information of the participants recruited.

Characteristics	Surgical treatment group (n=5)	Non-surgical treatment group (n=4)
Age, median (IQR)[a] years	85 (83, 88)	85.5 (84, 89.5)
Female, n (%)	5 (100%)	4 (100%)
Charlson Co-morbidity Index (CCI), median (IQR)	0 (0, 1)	0.5 (0, 1)
Montreal Cognitive Assessment (MoCA), median (IQR)	23 (16, 23)	24 (22, 29)*
Clinical Frailty Scale (CFS), median (IQR)	6 (4, 6)^	3 (2.5, 5)
Prescribed strong opioids, n(%)	5 (100%)	4 (100%)
Concomitant acute medical issues, n (%)	4 (80%)	2 (50%)
Presence of delirium, n (%)	0 (0%)	1 (25%)

*Data from 3 participants
^Data from 4 participants
[a] Inter-quartile range

For peer review only

Table 2. Outcomes at follow up visits compared to baseline measurement.

Characteristics: Median (IQR)[a]	Surgical treatment group (n=4)				Non-surgical treatment group (n=4)			
	Baseline	Week 2	Week 4	Week 12	Baseline	Week 2	Week 4	Week 12
Time up and go (TUG), measured in seconds	-	47.2 (29.9, 88.6)	-	22.6 (16.7, 25.1)	-	53.7 (28.0, 210.0)*	-	19.9 (19.0, 47.8)*
Roland Morris Disability Questionnaire (RMDQ)	-	13 (11.0, 15.5)	14.5 (9.5, 15.5)	8.5 (4.5, 10.5)	-	17.0 (14.0, 22.0)*	12.0 (10.0, 20.0)*	10.5 (6.0, 14.0)
Functional Independence Measure (FIM)	77.5 (67.5, 88.0)	114 (91, 119)*	-	120.5 (114.5, 125.0)	77.0 (51.5, 92.5)	100 (57, 117)	-	115.0 (86.0, 120.0)
Barthel Activities of Daily Living	11 (9, 13)	15.0 (11.5, 18.0)	19 (16, 19)*	19.0 (18.5, 19.5)	9 (5.5, 14.0)	14.0 (7.0, 17.0)	18.0 (14.0, 19.0)*	18.5 (11.5, 20.0)
Numeric pain rating scale	10 (9, 10)	5 (4.0, 7.0)*	5.0 (3.5, 6.5)	4.5 (2.5, 6.0)	10 (8, 10)*	7 (6.0, 8.0)*	7.0 (3.0, 9.0)*	4.5 (4.0, 5.0)†

*Data from 3 participants
†Data from 2 participants
[a] Inter-quartile range

For peer review only

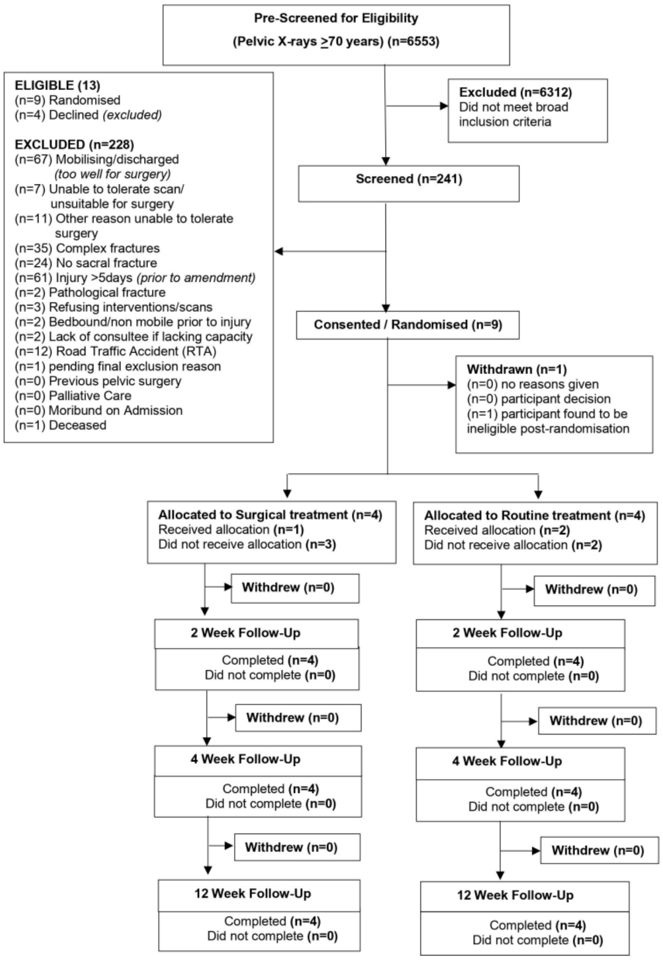

Figure 1. CONSORT diagram for the study.

CONSORT diagram for the study

209x297mm (300 x 300 DPI)

Reporting checklist for randomised trial.

Based on the CONSORT guidelines.

Instructions to authors

Complete this checklist by entering the page numbers from your manuscript where readers will find each of the items listed below.

Your article may not currently address all the items on the checklist. Please modify your text to include the missing information. If you are certain that an item does not apply, please write "n/a" and provide a short explanation.

Upload your completed checklist as an extra file when you submit to a journal.

In your methods section, say that you used the CONSORTreporting guidelines, and cite them as:

Schulz KF, Altman DG, Moher D, for the CONSORT Group. CONSORT 2010 Statement: updated guidelines for reporting parallel group randomised trials

		Reporting Item	Page Number
**Title and Abstract**			
Title	#1a	Identification as a randomized trial in the title.	1
Abstract	#1b	Structured summary of trial design, methods, results, and conclusions	1
**Introduction**			
Background and objectives	#2a	Scientific background and explanation of rationale	3
Background and objectives	#2b	Specific objectives or hypothesis	3
**Methods**			
Trial design	#3a	Description of trial design (such as parallel, factorial) including allocation ratio.	4
Trial design	#3b	Important changes to methods after trial commencement (such as eligibility criteria), with reasons	4

For peer review only

Participants	#4a	Eligibility criteria for participants	4
Participants	#4b	Settings and locations where the data were collected	4
Interventions	#5	The experimental and control interventions for each group with sufficient details to allow replication, including how and when they were actually administered	4
Outcomes	#6a	Completely defined prespecified primary and secondary outcome measures, including how and when they were assessed	4
Sample size	#7a	How sample size was determined.	4
Sample size	#7b	When applicable, explanation of any interim analyses and stopping guidelines	NA
Randomization - Sequence generation	#8a	Method used to generate the random allocation sequence.	
4			
Randomization - Sequence generation	#8b	Type of randomization; details of any restriction (such as blocking and block size)	
4			
Randomization - Allocation concealment mechanism	#9	Mechanism used to implement the random allocation sequence (such as sequentially numbered containers), describing any steps taken to conceal the sequence until interventions were assigned	NA
Randomization - Implementation	#10	Who generated the allocation sequence, who enrolled participants, and who assigned participants to interventions	4
Blinding	#11a	If done, who was blinded after assignment to interventions (for example, participants, care providers, those assessing outcomes) and how.	NA
Blinding	#11b	If relevant, description of the similarity of interventions	NA
Statistical methods	#12a	Statistical methods used to compare groups for primary and secondary outcomes	5
Statistical methods	#12b	Methods for additional analyses, such as subgroup analyses and adjusted analyses	NA

For peer review only

Outcomes	#6b	Any changes to trial outcomes after the trial commenced, with reasons	NA
**Results**			
Participant flow diagram (strongly recommended)	#13a	For each group, the numbers of participants who were randomly assigned, received intended treatment, and were analysed for the primary outcome	11
Participant flow	#13b	For each group, losses and exclusions after randomization, together with reason	11
Recruitment	#14a	Dates defining the periods of recruitment and follow-up	5
Recruitment	#14b	Why the trial ended or was stopped	NA
Baseline data	#15	A table showing baseline demographic and clinical characteristics for each group	12
Numbers analysed	#16	For each group, number of participants (denominator) included in each analysis and whether the analysis was by original assigned groups	12
Outcomes and estimation	#17a	For each primary and secondary outcome, results for each group, and the estimated effect size and its precision (such as 95% confidence interval)	5-6
Outcomes and estimation	#17b	For binary outcomes, presentation of both absolute and relative effect sizes is recommended	5-6
Ancillary analyses	#18	Results of any other analyses performed, including subgroup analyses and adjusted analyses, distinguishing pre-specified from exploratory	NA
Harms	#19	All important harms or unintended effects in each group (For specific guidance see CONSORT for harms)	5-6
**Discussion**			
Limitations	#20	Trial limitations, addressing sources of potential bias, imprecision, and, if relevant, multiplicity of analyses	6
Interpretation	#22	Interpretation consistent with results, balancing benefits and harms, and considering other relevant evidence	6
Registration	#23	Registration number and name of trial registry	5

For peer review only

Other Information

Protocol	#24	Where the full trial protocol can be accessed, if available	5
Funding	#25	Sources of funding and other support (such as supply of drugs), role of funders	8

The CONSORT checklist is distributed under the terms of the Creative Commons Attribution License CC-BY. This checklist was completed on 22. February 2021 using https://www.goodreports.org/, a tool made by the EQUATOR Network in collaboration with Penelope.ai

For peer review only
